# Gender Differences in Rates of Arrhythmias, Cardiac Implantable Electronic Devices, and Diagnostic Modalities Among Sarcoidosis Patients

**DOI:** 10.7759/cureus.7667

**Published:** 2020-04-14

**Authors:** Satya Durugu, Karthik Gonuguntla, Shivaraj Patil, Chaitanya Rojulpote, Vishruth Vyata, Pranav Karambelkar, Pranathi Narayanareddy, Kiranmayi Vuthaluru, Abhijit Bhattaru

**Affiliations:** 1 Internal Medicine, University of Louisville, Louisville, USA; 2 Internal Medicine, UConn Health Center, Farmington, USA; 3 Internal Medicine, University of Connecticut, Farmington, USA; 4 Internal Medicine, The Wright Center for Graduate Medical Education, Scranton, USA; 5 Nuclear Cardiology and Cardiovascular Molecular Imaging, University of Pennsylvania, Philadelphia, USA; 6 Internal Medicine, Osmania Medical College, Hyderabad, IND; 7 Internal Medicine, Hospital of the University of Pennsylvania, Philadelphia, USA; 8 Radiology, Hospital of the University of Pennsylvania, Philadelphia, USA

**Keywords:** sarcoidosis, arrhythmias, gender differences, implantable cardiac defibrillator, cardiac resynchronization therapy defibrillator

## Abstract

Introduction

Sarcoidosis is a granulomatous disease with multiorgan involvement. Cardiac involvement may be asymptomatic or present clinically as heart failure, arrhythmias, or even sudden cardiac death. In this study, we compared gender differences in the prevalence of arrhythmias and associated outcomes in patients with sarcoidosis without established coronary artery disease.

Methods

The United States Nationwide Inpatient Sample was queried from 2010 to 2014 to identify patients with sarcoidosis using the International Classification of Diseases, Ninth Revision (ICD-9) diagnosis code in patients >18 years. We excluded patients with a prior history of myocardial infarction, percutaneous coronary intervention, and coronary artery bypass graft. The chi-square test was used for statistical analysis.

Results

The sample consisted of 308,064 patients (mean age = 55.65 ± 11.28 years); they were mostly women (65.2%) and black (46.7%). In-hospital mortality in this cohort was 2.5%. The most common arrhythmia was atrial fibrillation (9.7%). The prevalence of ventricular fibrillation was 0.2%, ventricular tachycardia 2%, complete heart block 0.5%, and second-degree Mobitz type II (0.1%). Sudden cardiac death occurred in 0.7%. Rates of various cardiac devices implanted were: implantable cardiac defibrillator (ICD) (0.5%), cardiac resynchronization therapy-defibrillator (CRT-D) (0.2%), pacemaker (0.4%). Rates of endomyocardial biopsy (EMB), radionuclide imaging, and cardiac magnetic resonance imaging (MRI) were 0.2%, 0.3%, and 0.1%, respectively. Based on gender (male vs. female), the rates of arrhythmias, cardiac device implantation, and utilization of diagnostic modalities were: atrial fibrillation (41% vs 59%; p<0.001), ventricular fibrillation (50% vs 50%; p=0.983), ventricular tachycardia (55% vs 45%; p<0.001), complete heart block (48% vs 52%; p=0.3), second-degree Mobitz type II (37% vs 63%; p=0.706), sudden cardiac death (38% vs 62%; p<0.171), ICD (56% vs 44%; p<0.001), CRT-D (58% vs 42%; p=0.025), permanent pacemaker (40% vs 60%; p=0.066), EMB (55% vs 45%; p<0.001), radionuclide imaging (32% vs 68%; p=0.403), and cardiac MRI (41% vs 59%; p=0.396). In-hospital mortality was higher in females (64% vs 36%; p<0.001).

Conclusion

In our study, in-hospital death was more common in females. Females had higher rates of atrial fibrillation as compared to males, who were found to have a higher burden of ventricular tachycardia. Males had higher rates of ICD and CRT-D placement. Males also had EMB performed more commonly than females.

## Introduction

Sarcoidosis is a chronic granulomatous disease characterized by the presence of non-caseating granulomas constituting mononuclear phagocytes and T-lymphocytes infiltrate as a signature lesion. The etiologic agent has not been definitively identified and thus sarcoidosis remains a diagnosis of exclusion. This clinical entity may remain asymptomatic and subsequently be detected as an incidental finding, but most patients have a varied presentation, with symptoms appearing in different organ systems and ranging widely in severity and duration. It has been noted that those presenting with cardiac symptoms have some of the poorest outcomes. These cardiac symptoms most often manifest in the form of life-threatening arrhythmias and sudden cardiac death. It has been widely established that females are more prone to develop sarcoidosis than men [1-3]. Women with sarcoidosis are independently characterized by greater airflow obstruction, lower lung diffusing coefficient, older age, less smoking, and more frequent extrapulmonary complaints and musculoskeletal involvement [4].

This necessitates a more concerted approach when addressing female patients in both differential diagnostic and management settings. However, less is known about the gender distribution of the cardiac complications of sarcoidosis as compared to other extrapulmonary complications even though cardiac resonance magnetic imaging (MRI) and positron emission imaging (PET) have increasingly enabled their diagnosis. Using the National Inpatient Sample from 2010 to 2014, this study seeks to analyze the gender disparity in the prevalence of the disease, incidence of cardiac events, in-hospital mortality, rates of implantable cardiac defibrillator (ICD), cardiac resynchronization therapy defibrillator (CRT-D), and investigational procedures such as endomyocardial biopsy (EMB), cardiac magnetic resonance (CMR), and radionuclide imaging.

## Materials and methods

Data source

The National Inpatient Sample (NIS) database, which forms part of the Healthcare Cost and Utilization Project (HCUP), was queried from the years 2010 to 2014 and the data required were collected [5]. Having been initially created by the agency for healthcare research and quality maintenance, it now provides data on 5 to 8 million hospital stays from over 1,000 hospitals. To contain the margin for error of the estimates and make them more stable while at the same time increase precision, the database was designed to include data from a 20% sample of discharges from all participating hospitals. All participating states in the HCUP submit data to the NIS from their hospitals and this captures more than 95% of the US population. The database includes data from all non-federal, general, short-term, and other specialty hospitals in the United States (except rehabilitation and long-term acute-care hospitals) in the form of de-identified patient demographics, comorbidities, procedures, discharge diagnoses, outcomes, and total cost of hospitalization. Since the NIS database is available to the public and all patient information is anonymous, no approval was necessary from the local institutional review board (IRB).

Participant selection and covariates

The NIS was queried from 2010 to 2014 using the International Classification of Diseases, 9th Revision (ICD-9) diagnosis and procedure codes among patients 18 years or older. In our study, we utilized the ICD-9 diagnosis code 135 for sarcoidosis. All patients with a prior history of myocardial infarction, prior percutaneous coronary intervention/coronary artery bypass graft were excluded. Further, patients with incomplete data for gender and mortality were also excluded. To reduce the possibility of data duplication, we excluded patients who had an indication of transfer to another acute-care facility. The baseline characteristics of patients evaluated included age, sex, race, insurance, and hospital region. The complications associated with sarcoidosis and cardiac implantable device placements were identified using ICD-9 codes in the diagnosis fields and procedures performed were identified using ICD-9 codes in the procedure fields.

Statistical analysis

Differences among the categorical variables were tested using the chi-square test of independence or Fisher’s exact test. Patient characteristics (i.e., age, sex, and length of stay) and complications were entered in Table 1. Gender comparisons between the studied characteristics are displayed in Tables 2-3. A p-value ≤0.05 was taken as statistically significant.

**Table 1:** Characteristics of patients with sarcoidosis without CAD for index admission by year AF: atrial fibrillation; VF: ventricular fibrillation; VT: ventricular tachycardia; SCD: sudden cardiac death; CHB: complete heart block; MobitX2: Mobitz type 2 heart block; PPM: permanent pacemaker; EMB: endomyocardial biopsy; CMR: cardiac magnetic resonance; ICD: implantable cardiac defibrillator; CRT-D: cardiac resynchronization therapy defibrillator

Variable	Year					Total
	2010	2011	2012	2013	2014	
Total number of Sarcoid patients	65590	65204	59285	59085	58910	308,064
Mortality	1518 (2.3 %)	1717 (2.6 %)	1565 (2.6%)	1480 (2.5%)	1530 (2.6 %)	1574 (2.5%)
Age (years), M ± SD	54.54 ± 13.14	55.07 ± 13.29	55.97 ± 13.18	56.13 ± 13.34	56.66 ± 13.18	55.65 ± 13.25
Male	22032 (33.6%)	22707 (34.8%)	20355 (34.3%)	20680 (35%)	21520 (36.5%)	107294 (34.8%)
Race						
White	23967 (36.5%)	25281 (38.8%)	25020 (42.2%)	24885 (42.1%)	25420 (43.2%)	124573 (40.4%)
Black	31134 (47.5%)	30609 (46.9 %)	27730 (46.8%)	27750 (47%)	27000 (45.8 %)	144223 (46.8%)
Hispanic	2350 (3.6%)	2149 (3.2%)	1975 (3.3%)	2145 (3.6%)	2235 (3.8%)	10854 (3.5%)
Asian/Pacific Islander	311 (0.5 %)	238 (0.4%)	245 (0.4%)	230 (0.4%)	285 (0.5%)	1309 (0.4%)
Native American	185 (0.3 %)	208 (0.3 %)	200 (0.3 %)	150 (0.3%)	160 (0.3 %)	904 (0.3%)
Other	1011 (1.5%)	1164(1.8 %)	1595 (2.7 %)	1260 (2.1%)	1185 (2 %)	6215 (2%)
Unknown	6633 (10.3%)	5554 (8.7%)	2520 (4.3%)	2665 (4.5%)	2625 (4.5 %)	19996 (6.5%)
Insurance						
Medicare	26269 (40%)	27472 (42%)	26940 (45.4%)	27115 (45.9%)	27645 (46.9%)	135440 (43.9%)
Medicaid	10021 (15.3 %)	9923 (15.2%)	8310 (14%)	8825 (14.9%)	9150 (15.5%)	46229 (15%)
Private	23293 (35.6%)	22186 (34.1%)	19155 (32.3%)	18610 (31.5%)	18455 (31.3%)	101699 (33%)
Uninsured (self-pay)	3741 (5.7%)	3073 (4.7%)	2590 (4.4%)	2590 (4.4%)	1890 (3.2%)	13883 (4.5%)
Other	1877 (2.9%)	2158 (3.4%)	1925 (3.2%)	1445 (2.4%)	1295 (2.2%)	8700 (2.8%)
Unknown	389 (0.6%)	393 (0.6%)	365 (0.6%)	500 (0.8%)	475 (0.8%)	2122(0.7%)
Length of stay (days), M ± SD	4.92 ± 5.64	4.89 ± 5.34	4.93 ± 5.77	4.83 ± 5.133	4.91 ± 5.83	4.90 ± 5.542
Total charges (dollars), M ± SD	36032.31 ± 51334.36	38,550.46 ± 58302.15	41,296.46 ± 64836.62	43,633.34 ± 66768.93	46,106.77 ± 74,649.59	40,953.38 ± 63405.76
Complications:						
AF	5150 (7.9%)	6031 (9.3%)	5935 (10%)	6065 (10.3%)	6670 (11.3%)	29,850 (9.7%)
VF	156 (0.2%)	113 (0.2%)	145 (0.2%)	140 (0.2%)	115 (0.2 %)	669 (0.2%)
VT	1092 (1.7%)	1327 (2%)	1145 (1.9%)	1245 (2.1%)	1375 (2.3 %)	6184 (2%)
CHB	188 (0.3%)	263 (0.4%)	270 (0.5%)	375 (0.6%)	365 (0.6%)	1462 (0.5%)
MOBITX2	66 (0.1%)	20 (0.1%)	45 (0.1%)	60 (0.1%)	55 (0.1 %)	245 (0.1%)
SCA	409 (0.6 %)	365 (0.6%)	440 (0.7%)	430 (0.7%)	415 (0.7 %)	2059 (0.7%)
PPM	247 (0.4%)	227 (0.3%)	230 (0.4%)	305 (0.5 %)	250 (0.4 %)	1259 (0.4%)
ICD	313 (0.5 %)	324 (0.5%)	260 (0.4%)	260 (0.4%)	295 (0.5 %)	1452 (0.5%)
CRT-D	106 (0.2%)	122 (0.2%)	80 (0.1%)	115 (0.2%)	130 (0.2 %)	553 (0.2%)
Radionuclide Scans	174 (0.3 %)	205 (0.3%)	180 (0.3%)	185 (0.3%)	135 (0.2 %)	879 (0.3%)
EMB	101 (0.2%)	78 (0.1%)	85 (0.1%)	105 (0.2 %)	100 (0.2 %)	470 (0.2%)
CMR	65 (0.1 %)	49 (0.1%)	70 (0.1 %)	10 (0.02%)	30 (0.1%)	224 (0.1%)

**Table 2:** Gender differences in rates of arrhythmias and diagnostic and interventional procedural rates in patients of sarcoidosis AF: atrial fibrillation; VF: ventricular fibrillation; VT: ventricular tachycardia; SCD: sudden cardiac death; CHB: complete heart block; MobitX2: Mobitz type 2 heart block; PPM: permanent pacemaker; EMB: endomyocardial biopsy; CMR: cardiac magnetic resonance; ICD: implantable cardiac defibrillator; CRT-D: cardiac resynchronization therapy defibrillator

SEX.	2010	2011	2012	2013	2014	All years combined
0=Male						
1=Female						
AF	(p<0.01)	(p<0.01)	(p<0.01)	(p<0.01)	(p<0.01)	(p<0.001)
0=Male	40%	43%	39%	40%	43%	41%
1=Female	60%	57%	61%	60%	57%	59%
VF	(p=0.983)	(p<0.001)	(p=0.113)	(p=0.039)	(p=0.119)	(p=0.983)
0=Male	33%	67%	48%	54%	36%	50%
1=Female	67%	33%	52%	46%	64%	50%
VT	(p<0.01)	(p<0.01)	(p<0.01)	(p<0.01)	(p<0.01)	(p<0.01)
0=Male	53%	54%	54%	55%	60%	55%
1=Female	47%	46%	46%	45%	40%	45%
CHB	(p=0.300)	(p=0.05)	(p=0.015)	(p=0.018)	(p=0.123)	(p=0.3)
0=Male	42%	53%	50%	46%	45%	48%
1=Female	58%	47%	50%	54%	55%	52%
MOBITX2	(p=0.706)	(p=0.524)	(p=0.142)	(p=0.904)	(p=0.214)	(p=0.706)
0=Male	38%	50%	11%	33%	55%	37%
1=Female	62%	50%	89%	67%	45%	63%
SCD	(p=0.965)	(p=0.710)	(p=0.859)	(p=0.043)	(p=0.701)	(p=0.171)
0=Male	34%	37%	35%	45%	38%	38%
1=Female	66%	63%	65%	55%	62%	62%
PPM	(p=0.897)	(p=0.845)	(p=0.492)	(p=0.211)	(p=0.047)	(p=0.066)
0=Male	33%	36%	39%	43%	50%	40%
1=Female	67%	64%	61%	57%	50%	60%
ICD	(p<0.01)	(p<0.01)	(p<0.01)	(p=0.048)	(p<0.01)	(p<0.001)
0=Male	34 57%	48 62%	31 60%	25 48%	35 59%	56%
1=Female	26 43%	30 38%	21 40%	27 52%	24 41%	44%
CRT-D	(p=0.012)	(p=0.166)	(p=0.001)	(p=0.030)	(p=0.025)	(p=0.025)
0=Male	60%	48%	75%	56%	58%	58%
1=Female	40%	52%	25%	44%	42%	42%
Radionuclide scans	(p=0.982)	(p=0.111)	(p=0.407)	(p=0.986)	(p=0.393)	(p=0.403)
0=Male	33%	23%	28%	35%	44%	32%
1=Female	67%	77%	72%	65%	56%	68%
EMB	(p<0.01)	(p=0.010)	(p=0.033)	(p=0.536)	(p=0.086)	(p<0.001)
0=Male	73%	65%	59%	29%	55%	55%
1=Female	27%	35%	41%	71%	45%	45%
CMR	(p=0.015)	(p=0.099)	(p=0.502)	(p=0.656)	(p=0.871)	(p=0.396)
0=Male	67%	10%	43%	50%	33%	41%
1=Female	33%	90%	57%	50%	67%	59%
Race:	Whites	Whites	Whites	Whites	Whites	Whites
Male vs	37% vs	37% vs	37% vs	37% vs	39% vs	(37% vs
female	63%	63%	63%	63%	61%	63%)
p-value	p<0.01	p<0.01	p<0.01	p<0.01	p<0.01	p<0.01
Race:	Black	Black	Black	Black	Black	Black
Males vs	31% vs	33% vs	31% vs	33% vs	34% vs	(48% vs
Female	69%	67%	69%	67%	66%	52%)
p-value	p< 0.01	p<0.01	p<0.01	p< 0.01	p<0.01	p< 0.01

**Table 3:** Gender differences in the in-hospital mortality rates among patients of sarcoidosis

p-value	(p=0.042)	(p=0.655)	(p=0.766)	(p=0.002)	(p=0.097)	(p<0.001)
0=Male	29%	34%	33%	44%	32%	36%
1=Female	71%	66%	67%	56%	68%	64%

## Results

The NIS was queried from 2010 to 2014 and yielded 308,064 cases of sarcoidosis with a mean age of 55.65 + 11.28 years. Women constituted 65.2% of the total sample size and men constituted 34.8%. When analyzed for gender differences for the rates of arrhythmias for the years 2010 to 2014, atrial fibrillation (AF) was found to be higher in females (41% in males vs 59% in females, p<0.001), ventricular fibrillation (VF) was comparable (50% in males vs 50% in females, p=0.983), ventricular tachycardias (VT) were higher in males (55% in males vs 45% in females, p<0.001), complete heart blocks were higher in females (48% in males vs 52% in females, p=0.3), and Mobitz type II blocks were higher in females (37% in males vs 63% in females, p=0.706) as well. When analyzed for the incidence of sudden cardiac death, the rates among females were found to be higher (38% in males vs 62% in females, p=0.171).

The rates of cardiac MRI were higher in females (41% in males vs 59% in females, p=0.396) and the rates of radionuclide scans were also higher in females (32% in males vs 45% in females, p=0.403). Interestingly, more males required an endomyocardial biopsy (55% in males vs 45% in females, p<0.001).

Our study also looked at the procedural rate required by the cohort and the results yielded showed that men were more likely to require an implantable cardioverter-defibrillator (56% in males vs 44% in females, p<0.001) and cardiac resynchronization therapy-defibrillator (58% in males vs 42% in females, p=0.025). However, pacemaker insertion rates were higher in females (40% in males vs 60% in females, p=0.066).

## Discussion

Sarcoid lesions are most frequently present in the lung but can affect multiple organ systems, with 30% of the patients presenting with extra-pulmonary symptoms, with cardiac involvement carrying a significantly poorer outcome in terms of all-cause mortality [6-9]. Our study found that among both gender groups, females had a higher prevalence of sarcoidosis (65.2%) as compared to males (34.8%). This is in accordance with other studies that have found females to have a higher incidence and prevalence of sarcoidosis as compared to males [1-3]. Similarly, Rybicki et al. reported that there was an overall increased risk of developing sarcoidosis with an unadjusted relative risk of 1.43 (95% CI 1.11-1.84), which fell to 1.3 after adjusting for demographic factors, highlighting the importance of demographics in this disease [2]. Women were also found to have a higher mean age at diagnosis and an increased risk of developing extra-pulmonary complications [10]. An analysis of racial differences revealed that patients of African-American origin had the highest prevalence of sarcoidosis (46.8%) as compared to any other race. Interestingly, a study published by Benjamin et al. found that the higher occurrence of sarcoidosis among females was also observed among African-American females, with an incidence of 39.1 (95% CI 29.4-48.8) in comparison to their male counterparts who registered 29.8 (95% CI 19.2-40.4) [2]. Similarly, Caucasian females had a higher incidence rate of 12.1 (95% CI 8.2-16.1) as compared to only 9.6 (95% CI 5.8-13.3) in Caucasian males [2].

The most common complication observed in this population is an atrioventricular block (AV), with an initial presentation of a PR prolongation that subsequently progresses to a complete AV block [11-12]. This is in contrast to our study where atrial fibrillation (AF) was the most common complication (9.7%). Moreover, the incidence of arrhythmia was found to be higher in females (59% females vs 48% in males, p<0.001). In fact, the presence of sarcoidosis alone increases the risk for females to develop AF to higher rates than observed in the general population of comparable age group (23.2%, 95% CI 21.3-24.3) [13]. Our study also found that after AF, ventricular tachycardia (VT) was the most prevalent arrhythmia in this population (2%). This is in accordance with previously established data [11]. Moreover, VT was found to have a higher incidence in males compared to females (55% vs 45%, p<0.001). With regards to the rates of ventricular fibrillation in this population, we found that both gender groups were comparable (50% males vs 50%, females, p=0.983).

A complete heart block (CHB) was found in 0.5% of patients. This low figure may be explained by the fact that a third-degree AV block has a higher prevalence in younger patients (mean age of our study was 55.65 years), as the pathogenesis of sarcoidosis is different compared to other etiologies [14]. When analyzed based on gender, the difference found in our data was not significant in CHB (48% in males vs 52% in females, p<0.3). Sudden cardiac death (SCD) occurred in 0.7% of patients, of which many may be attributable to either AV block or VT degenerating into VF. A comparison of SCD between both gender groups was not found to be significant (38% males vs 62% females, p<0.171). This may be attributed to the fact that ventricular arrhythmias have a comparable number of females and males.

When sarcoidosis is suspected, it is imperative to conduct timely investigations that allow for a definitive diagnosis and appropriate management. Cardiac magnetic resonance imaging (CMR) is the technique of choice in the evaluation of patients with suspected sarcoid involvement of the heart, with a high negative predictive value and sensitivity [15-16]. A higher number of females required a diagnostic CMR as compared to males (59% females vs 41% males; p=0.396). Due to the low positive predictive value, it is important to follow up with confirmatory testing such as endomyocardial-biopsy. Endomyocardial-biopsy (EMB) is the gold standard with high specificity for detecting sarcoid lesions in the heart. However, the procedure has a low sensitivity approaching 20% in a study done on a series of 26 patients [17]. A higher percentage of males were found to require an EMB when compared to females (55% males vs 45% females, p<0.001). Our study found that 0.3% of individuals received radionuclide scans, with females having undergone the investigation more frequently than males (68% vs 32%, p=0.403). Though the role of radionuclide scans, namely, positron emission tomography (PET) scan, is widely used for cancer detection, it has gained increasing popularity in recent years for cardiovascular purposes [18-24].

Patients who have a sustained VT or VF are recommended ICD placement. More males required an ICD compared to females (56% vs 44%, p<0.001). This may be explained by the observed higher incidence of VT in males. Similarly, CRT-D placement was also found to be higher in males than females (58% vs 42%, p<0.025). The significance of these values can be collaborated with another study by Lin et al. where males had a higher total age-adjusted incidence of ICD placement of 42.63 (95% CI 34-51.30). This finding has also been observed across all age-groups [25]. There has been an increasing trend in pacemakers in recent years [26]. Our study has found that pacemaker insertion rates were higher in females (40% in males vs 60% in females, p=0.066).

Despite the greater procedural interventions required by males, our study shows that women with adverse cardiac events face poorer clinical outcomes as evidenced by the higher in-hospital mortality rates (64% in females vs 36% males, p <0.001). This increased mortality depicts gender as an independent risk factor to the poor outcomes seen in sarcoidosis. This is in line with unfavorable outcomes in females with pulmonary involvement and may be due to a greater obstruction to airflow, older age of incidence, lower coefficient of lung diffusion, and more frequent extrapulmonary complaints [4]. Few studies have been done to address this gender disparity, with little conclusive evidence. A possible explanation may be found in the study by Chioma et al., which has found that the cell cycle inhibitory molecule programmed death-1 is significantly upregulated on CD4+ T cells in females with sarcoidosis, which promoted fibrotic reactions, thus causing fibrosis-related cardiac complications in this population (Abstract: Chioma OS, Celada LJ, Abel K, Newcomb DC, WP Drake. Gender Differences in Sarcoidosis Mediated by PD-1, and TH17 Pathway. ATS 2019; May 19/2. https://www.atsjournals.org/doi/10.1164/ajrccm-conference.2019.199.1_MeetingAbstracts.A2432).

## Conclusions

It is important to recognize the role of gender, as it plays a crucial role in the incidence and prognosis of cardiac events in sarcoidosis. Females have a higher preponderance for in-hospital mortality in relation to adverse cardiac complications in relation to sarcoidosis. Recognizing the role of demographics like gender and race may help in identifying sarcoidosis individuals who may be at a higher risk of adverse events.
